# Realization of Three-Dimensionally MEMS Stacked Comb Structures for Microactuators Using Low-Temperature Multi-Wafer Bonding with Self-Alignment Techniques in CMOS-Compatible Processes

**DOI:** 10.3390/mi12121481

**Published:** 2021-11-29

**Authors:** Adrian J. T. Teo, King Ho Holden Li

**Affiliations:** School of Mechanical and Aerospace Engineering, Nanyang Technological University, 50 Nanyang Avenue, Singapore 639798, Singapore; Adrian.teojt@ntu.edu.sg

**Keywords:** 3D MEMS, wafer bonding, CMOS–MEMS compatibility, multiple wafer stacking, alignment error corrections

## Abstract

A high-aspect-ratio three-dimensionally (3D) stacked comb structure for micromirror application is demonstrated by wafer bonding technology in CMOS-compatible processes in this work. A vertically stacked comb structure is designed to circumvent any misalignment issues that could arise from multiple wafer bonding. These out-of-plane comb drives are used for the bias actuation to achieve a larger tilt angle for micromirrors. The high-aspect-ratio mechanical structure is realized by the deep reactive ion etching of silicon, and the notching effect in silicon-on-insulator (SOI) wafers is minimized. The low-temperature bonding of two patterned wafers is achieved with fusion bonding, and a high bond strength up to 2.5 J/m^2^ is obtained, which sustains subsequent processing steps. Furthermore, the dependency of resonant frequency on device dimensions is studied systematically, which provides useful guidelines for future design and application. A finalized device fabricated here was also tested to have a resonant frequency of 17.57 kHz and a tilt angle of 70° under an AC bias voltage of 2 V.

## 1. Introduction

The term “plenty of room at the bottom” [1] is synonymous with the birth of the complementary metal–oxide–semiconductor (CMOS), with exponential growth in the last six decades for the semiconductor industry. Micro-electro-mechanical system (MEMS) sensors were built upon the success of the CMOS industry and its infrastructures. In recent years, nano-electro-mechanical systems (NEMS) have also attracted much attention due to the possibility of observing quantum effects in systems much larger than a few atom ensembles. Some of the success of MEMS sensors includes accelerometers for airbag application, inertia sensors for navigation and consumer electronics, timing devices, biosensors and many other products that are manufactured and shipped in large commercial volumes and produced in foundries. Often seen as the late comer or the little cousin of CMOS, researchers and industries are always aiming towards the realization of integrating both MEMS and CMOS on a single platform [2,3] for the most advanced applications, such as military or high-end consumer products.

Unlike CMOS fabrication processes and the constant size shrinkage of transistors under Moore’s law, the demand of high-performance MEMS devices is often multi-faceted. In the case of inertia sensors, MEMS sensors are required to have a larger proof mass [4]. On the other hand of radio frequency (RF) MEMS application, there is a need to create MEMS structures as inductor coils [5]. Mastering the process of multi-stack silicon-direct bonding processes, a six-wafer combustion system has been demonstrated for MEMS manufacturing [6]. In all cases, the common theme is gearing towards a three-dimensional (3D) MEMS fabrication process that is versatile and easy to be adopted by foundries for mass manufacturing. Often, the ability to integrate both CMOS and MEMS on a single platform would offer other potential benefits towards high-performance sensors [7].

Another difficulty of achieving a high level of acceptance of 3D MEMS lies in the alignment error introduced during the wafer bonding process. In the case of conventional comb drives with a few micrometers of width for capacitive sensing devices, a slight misalignment of two micrometers, not including the rotational error, would render the device useless. Herein lies the interest in demonstrating a potential capability of fabricating 3D MEMS devices, such as actuators, on CMOS-compatible multiple wafer stacking technologies. This would reduce any alignment issues that could affect the overall performance of the final product, hence maintaining a high production yield. Current state-of-the-art methods involve using a pre-fabricated CMOS chip as a base substrate to fabricate the MEMS layered devices [3,8]. This, however, further extends the fabrication process instead of amalgamating it, leading to a higher risk of errors and defects. 

For the past decade, a growing demand has emerged for a high-speed, high-resolution and high-quality MEMS actuator for various applications. Some examples of these are the LIDAR for autonomous driving [9], optical fiber switch [10] and optical scanning [11]. Most of such electrostatic actuators can be classified under two categories in terms of their moving directions, i.e., in-plane and out-of-plane. In the case of comb-drive actuators, there are lateral comb-drive actuators [12] and vertical comb-drive actuators [13]. The vertical comb-drive actuator is found particularly useful in scanning micromirror applications [11] and is regarded as robust and suitable for 3D micromachining for obtaining a large tilt angle.

High-aspect-ratio-micromachining processes, such as deep reactive ion etching (DRIE), have enabled the realization of MEMS devices with even larger angular motion [14,15]. Uma et al. have demonstrated a 3D stacked micromirror with an improved performance of tilt angle of about 40° at resonant frequency [16]. In addition to DRIE, a fusion bonding process that is CMOS–MEMS compatible is another essential process step for 3D microsystems fabrication. To this end, Tan et al. have developed a low-temperature (<300 °C) fusion bonding process using different intermediate dielectric layers [17,18]. Kühne et al. further applied the low-temperature wafer bonding technology for the fabrication of high-aspect-ratio MEMS [19]. Low-temperature bonding is essential to carefully control the amount of thermal loading on the wafers prior to stacking, as these wafers already have MEMS devices and interconnect on them. Extrinsic thermal stress developed during cooling due to the coefficient of thermal expansion mismatch of the multi-layer stack would thus be reduced. Simultaneously, this bonding process also reduces the risk of misalignment due to the thermal reflow of the bonding agent or ultrasonic vibration during state-of-the-art thermal and ultrasonic bonding processes. The above development has further enabled the realization of 3D MEMS with vertical comb drives for a range of potential applications.

This work presented here combines the design and demonstration of a high-aspect-ratio 3D MEMS vertical comb structure, where a novel low-temperature bonding process of two patterned wafers is achieved for the first time. This is a key processing method to realize the 3D vertical comb structures. The fabrication process also reveals the ease and capability of fabricating 3D MEMS actuators on CMOS-compatible technologies, as mentioned earlier. The device and process flow design are discussed first, followed by the fabrication results. Finally, a systematic study on the resonant frequency of the 3D MEMS devices is also presented that is CMOS-compatible. The 3D MEMS devices have been designed to have self-aligned vertical comb-drive structures, with the back-side alignment being the key feature of this fabrication process. In order to generate a high electrostatic torque with a large tilt angle, vertical comb drives with a high-aspect-ratio profile (>40 µm deep, 6um width fingers) have been fabricated by DRIE technology. With total freedom in three dimensions with these stacking structures, the devices are expected to achieve a higher tilt angle than the conventional 2D planar devices.

## 2. Device and Process Flow Design

**Figure 1:**
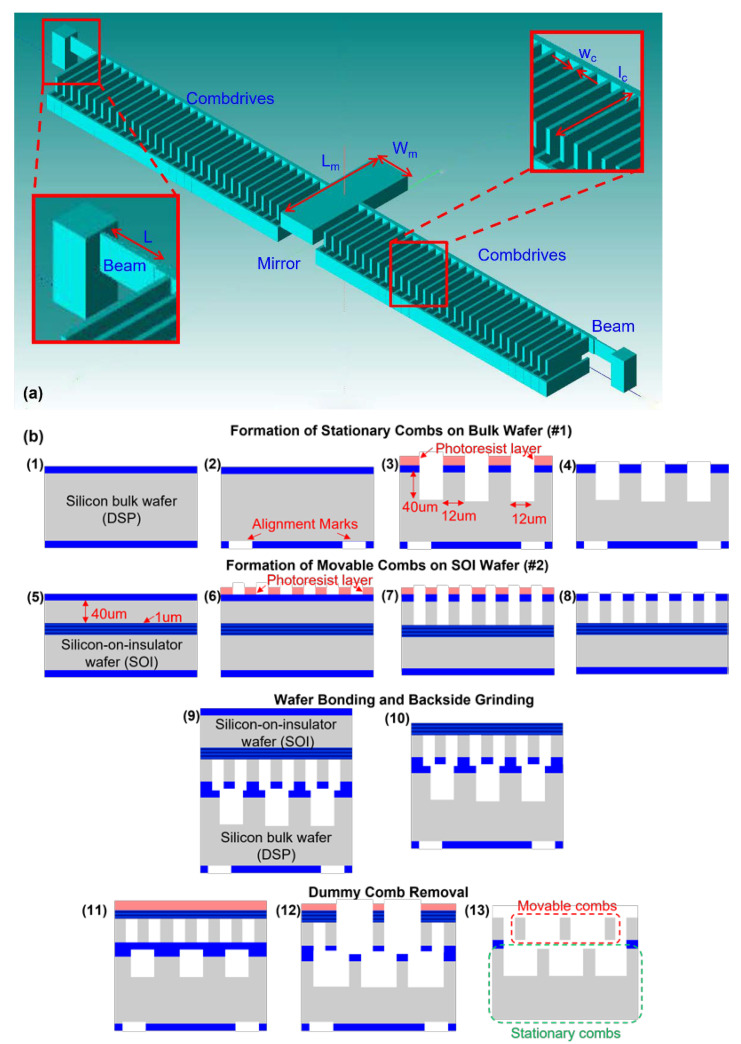
Schematic of (**a**) the 3D comb drive structure and (**b**) process flow of the CMOS-compatible fabrication process.

The schematic diagram of the device designed for this work is shown in Figure 1a. The 3D comb drive has two arrays of fingers stacked vertically (i.e., an array of stationary bottom combs and an array of movable top combs), which can generate out-of-plane displacement including torsion and piston motion. Table 1 provides the key design parameters of one standard device with dimensions and nominal values. Devices with different beam dimensions and mirror sizes are designed to provide variations in generating datasets for device optimization, which will in turn provide valuable information on the fabrication limitations for the designers.

The process flow with the four main steps is shown in Figure 1b. Firstly, as shown in steps 1–4, the stationary combs are patterned and etched with DRIE on the bottom bulk Si (double-side polished) using thermal oxide (SiO_2_) as the hard mask. The purpose of having back-side alignment marks included on this wafer is to facilitate front-to-back alignment in the subsequent steps. Secondly, the movable combs together, with the beam and mirror, are fabricated on the device layer of the SOI wafer, as shown in steps 5–8. Note that there are two sets of fingers on the SOI wafer. Besides the actual device fingers that eventually form the top movable comb, dummy fingers are inserted alternately in between these device fingers as a sacrificial mask to trim the larger fingers on the bottom wafer (more details will be shared in the next section). Thirdly, as shown in steps 9–10, these two patterned wafers are aligned face-to-face (using the back-side alignment marks of the bottom wafer and the front-side alignment marks of the top wafer) and bonded using the low-temperature wafer bonding method. The substrate of the top SOI wafer is finally thinned down and stopped selectively on the buried oxide (BOX) layer to release the movable combs.

A lithography step is performed on the BOX layer, as given in steps 11–13. The dummy fingers are sacrificially removed by DRIE etching, and this process trims the larger finger on the bottom wafer. This would thus be the crucial self-alignment step, as praised in the earlier sections. As the etching of both the SOI and bulk wafer to reveal the comb fingers is carried out using a single mask, no alignment is required for the fingers on each substrate. The two sets of comb fingers are spaced precisely apart and the overlay error during wafer-to-wafer alignment is essentially eliminated. At the same time, the contact pads of the bottom wafers are formed by DRIE etching through the top wafer to the bottom wafer. A schematic view of the final 3D comb structure is given in step 13. This CMOS-compatible process is used throughout the whole process, which will be useful for the future application of CMOS–MEMS integration.

## 3. Fabrication Results

### 3.1. Wafer Bonding

**Figure 2:**
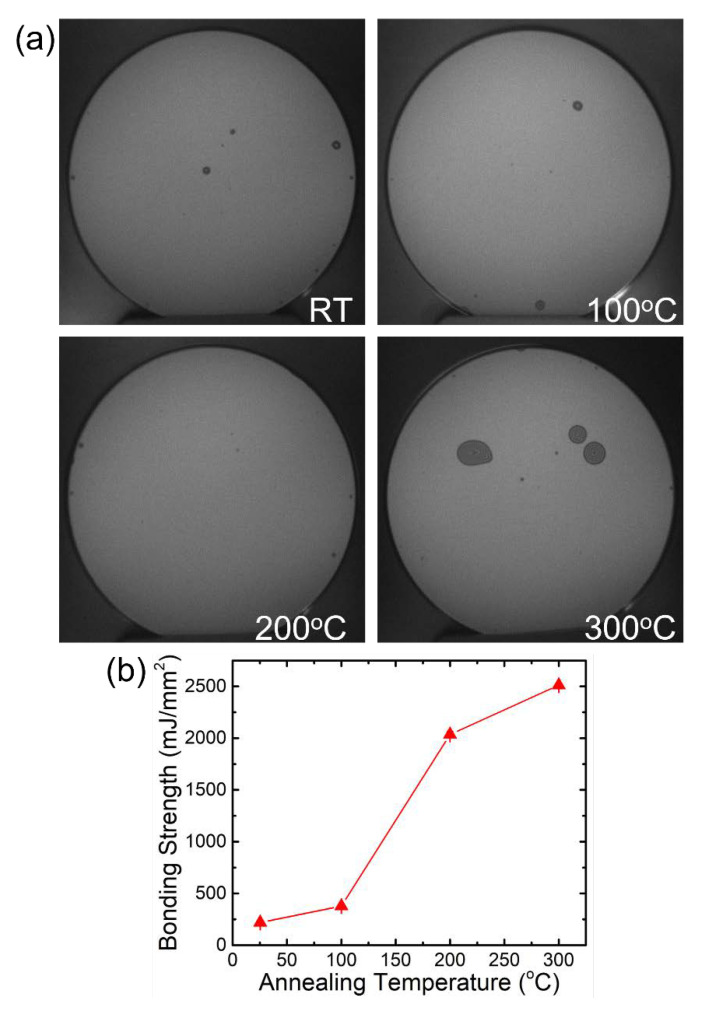
(**a**) IR images for Si-to-Si wafer bonding annealed at different temperatures. Random voids are observed to have greater influences at higher temperatures and (**b**) the corresponding bond strength measurement at the different temperatures.

One of the important processes in 3D MEMS device fabrication is wafer bonding, by which the vertical combs are fabricated in a different plane, with the aim of achieving a larger tilt angle. Our wafer bonding studies start from basic wafer bonding between two blanket wafers. The wafers used in this experiment are p-type 150 mm Si (100) test wafers with a resistivity in the range of 4–10 Ω∙cm. In the Si–Si wafer fusion bonding experiment, the high-energy hydrophilic surfaces are formed by exposing the wafers to O_2_-plasma for 15 s and bonded at room temperature on a commercial double-side aligner. These steps promote the surface conditions for successful wafer bonding at low temperatures. After bonding, the bonded wafer pairs are annealed at a temperature range of 100–300 °C in an atmospheric N_2_ ambient for 3 Hr to further enhance the bond strength. IR transmission images of the bonded wafers are shown in Figure 2a. This bonding method was previously used for hermetic packaging in our previous work, as cited here [20].

The bonding strength is determined by Maszara’s crack-opening method, using a blade and IR imaging system. Our experimental results show that the bonding strength increases with the annealing temperature as expected, and the bonding strength approaches the silicon fracture energies (~2.5 J/m^2^) at an annealing temperature of 300 °C, as shown in Figure 2b. Some random voids are observed in the IR images due to unintentionally trapped particles during wafer transfer and handling.

**Figure 3:**
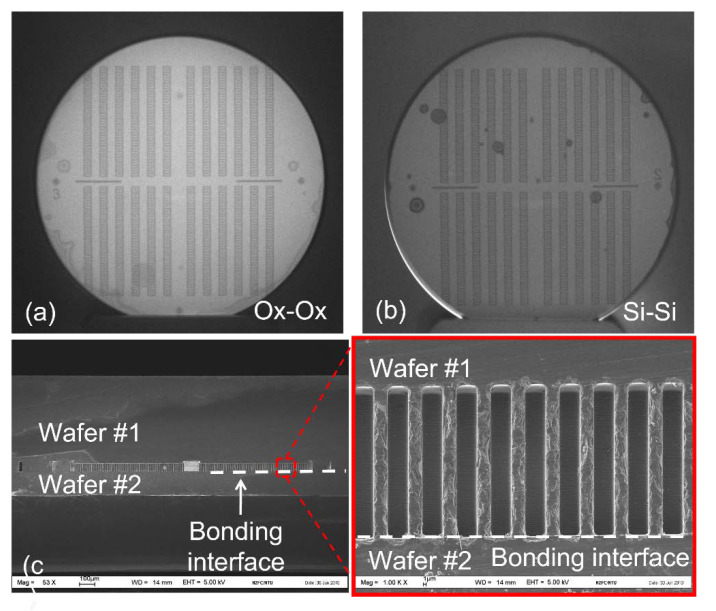
IR images for (**a**) oxide-to-oxide and (**b**) Si-to-Si wafer bonding using one blanket wafer and (**c**) one patterned wafer and cross-sectional SEM image for oxide–oxide wafer bonding.

Wafer bonding between a patterned/etched wafer and a blanket wafer is investigated based on the results of the blanked wafer bonding. The same process conditions, including plasma activation, are used for this experiment. IR images of the bonded wafers with and without the oxide layer are shown in Figure 3a,b, respectively. Both IR images show the bonding interface without any visible bubble. Since it is difficult to obtain the bonding strength by Maszara’s crack-opening method for the patterned wafer, scanning electron microscopy (SEM) is a better choice to observe the bonding interface and bonding quality. The bonded wafer pair is partially diced, and the bonding interface is examined in SEM. A cross-sectional SEM image of the bonded wafer pair is shown in Figure 3c, showing a defect free interface.

**Figure 4:**
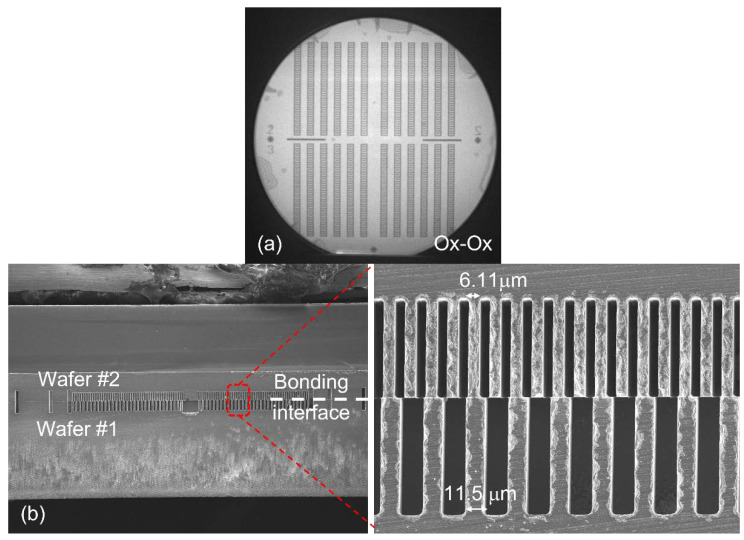
IR images for (**a**) oxide-to-oxide wafer bonding using two patterned wafers with a 40 µm deep trench and (**b**) cross-sectional SEM image of the bonded wafers.

With the promising results given in Figure 3, there is a need to verify oxide–oxide bonding using actual patterned wafers. Two patterned wafers with a 40 µm deep trench were used to investigate the bonding quality for actual production. Each wafer has the etch pattern of the top and bottom wafers in the actual 3D MEMS process flow. Both wafers are double-side polished 150 mm p-type Si (100) wafers. The IR and SEM images of the bonded pair are shown in Figure 4. From the bonding results, the two wafers are held by the bonded thermal oxide layer (200 nm on each wafer), and it is mechanically robust to sustain the shear force during wafer dicing. It is also observed that the dummy fingers (alternate ones) of the top wafer are bonded to the larger fingers in the bottom fingers. The above studies establish the baseline bonding process for subsequent device fabrication.

### 3.2. Device Fabrication

As described earlier in Figure 1, the 3D MEMS devices are built from two bonded wafers (150 mm), one double-side polished (DSP) wafer containing the stationary combs and an SOI wafer (40 µm SOI, 1 µm BOX, 500 µm substrate) containing the movable combs at the upper plane. The SOI wafer is an n-type doped prime-quality wafer with a very low resistivity of less than 0.005 Ω∙cm. The BOX layer is used as the selective etch stop layer in order to achieve a uniform plane for the movable fingers. Therefore, the application of an SOI platform is very attractive for integrating MEMS devices and electronics to create a 3D microsystem.

The DSP and SOI wafers are first cleaned using a standard RCA1 solution (NH_4_OH:H_2_O_2_:H_2_O = 1:1:5 at 80 °C for 10 min) to remove the organic contaminants, followed by a short immersion in dilute HF for oxide stripping. The cleaning ends with an RCA2 solution (HCl:H_2_O_2_:H_2_O = 1:1:6 at 80 °C for 10 min) to remove the metallic contaminants. The wafers are spin-dried and thermal oxide (wet process) of 2000 Å is grown on the wafers at 1000 °C. In this process, the chemical mechanical polishing (CMP) process, which is commonly used to improve the surface roughness to promote bonding, is not necessary since the surface roughness is kept at <1 nm by the virtue of the thermal oxidation used on this work. On the other hand, CMP is needed when the oxide layer is deposited via the chemical vapor deposition (CVD) method. This point is reaffirmed as the wafers used for bonding, as shown in Figure 2, Figure 3 and Figure 4, are without any surface polishing.

**Figure 5:**
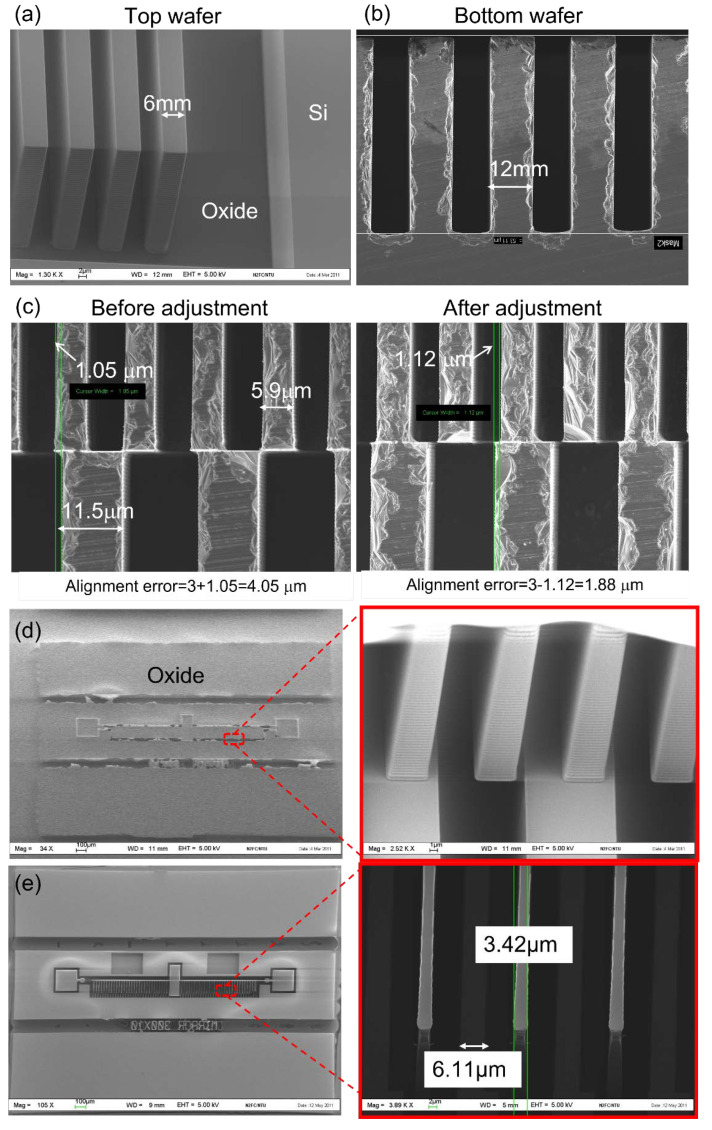
SEM images for (**a**) SOI wafers, (**b**) DSP wafers, (**c**) misalignment estimation after wafer bonding, (**d**) after back-side thinning and (**e**) final fabricated device.

On top of wafer bonding, another important process step is Si etching by DRIE using a Bosch cyclic etch-passivation sequence. The width of the top comb fingers is just 6 µm, so an etch profile as close to 90° as possible is critical. In NTU’s cleanroom, the DRIE tool from SPP is used to process the comb structures, and the desired comb fingers (6 µm-width, 40 µm-depth and 6 µm-spacing) are achieved. The etch profile is shown in Figure 5. In Figure 5a, the SEM image is taken from the top SOI wafer showing clear comb structures with negligible notching at the bottom after the DRIE process, and the finger width is close to 6 µm. Similarly, in Figure 5b, the SEM image shows the 12 µm wide comb finger from the bottom DSP wafer. The larger fingers are trimmed to 6 µm, subsequently using the top sacrificial dummy fingers. After the wafers are etched based on the desired design, wafer bonding is carried out based on the process steps described in the earlier section.

During the pre-bonding stage, keeping the wafer-to-wafer misalignment as small as possible is a very critical consideration, since patterned/etched wafers are used for bonding. This is controlled carefully by investigating the machine limitation, changing the proximity flags and adjusting the pre-bonding parameters. As can be seen in Figure 5c, an improvement in misalignment error from 4.05 µm down to 1.88 µm has been achieved using the above precautions and thus making the device fabrication possible. Based on our design, a maximum misalignment of 3.0 µm can be tolerated to ensure that the top and bottom fingers are placed properly in the final 3D comb stack.

After wafer bonding and annealing, back-side grinding is subsequently performed and a bond strength of >1 mJ/m^2^ has held the bonded wafer pair without detachment despite the high shear force during mechanical grinding. The result is given in Figure 5d. The next critical step is the photolithography step on the BOX “membrane” and great care must be exercised to prevent potential damage to the BOX layer. Despite this challenge, a photo-resist mask is successfully applied and patterned, followed by oxide breaking and Si etching, all carried out using plasma etching. During this etching step, the top device fingers are protected by the photo-resist mask, while the sacrificial dummy fingers are exposed and completely etched. These dummy fingers are designed to be smaller (~6 µm) than the fingers on the bottom DSP wafers (12 µm), in order to allow trimming of the bottom fingers to 6 µm. As presented in Figure 5e, bottom fingers of 6.1 µm are successfully trimmed from the initial width of 12 µm after the trimming step. The remaining top moveable fingers are measured to be 3.42 µm (as opposed to the intended 6 µm in the design), and this is attributed to misalignment in the final lithography step and undercutting during the etching step. Despite this, a vertical stack consisting of two out-of-place comb structures is fabricated and ready for testing.

## 4. Device Performance

The resonant frequency of a rectangular mirror in torsion mode can be expressed as:

(1) $$f_{0}=\frac{1}{2\pi }\sqrt{\frac{K_{\theta }}{I_{\theta }}}\;\;\;\;\;\;$$

where $K_{\theta }$ is the stiffness of the beam and $I_{\theta }$ is the torsional moment of inertia for micromirror rotation. They can be calculated by [21,22]:

(2) $$K_{\theta }=\frac{G}{L}ab^{3}\left[\frac{16}{3}-3.36\frac{b}{a}\left(1-\frac{b^{4}}{12a^{4}}\right)\right]\;\left(a>b\right)\;\;\;\;\;\;\;\;\;\;\;\;\;\;\;\;\;\;\;\;\;\;\;\;\;\;$$

(3) $$I_{\theta }=\rho t\left\{\frac{L_{m}\cdot W_{m}^{3}}{12}+N\left[\frac{w_{c}\cdot l_{c}^{3}}{12}+w_{c}\cdot l_{c}{\left(\frac{w_{c}+l_{c}}{2}\right)}^{2}\right]\right\}\;\;\;\;\;\;\;\;\;\;\;\;\;\;\;\;\;\;\;\;\;\;$$

where ρ is the density of the mirror material, t is the thickness of mirror plate, $L_{m}$ and $W_{m}$ are the length and width of mirror plate, respectively, $l_{c}$ and $w_{c}$ are the length and width of the comb finger, respectively, N is the number of comb fingers, L is the length of the beam and a and b are the half length of the long side and short side of the two sides of the cross-section of the beam. Here, a equals to t, and b is the width of the beam. G is the shear modulus of silicon. The resonant frequency of the 3D comb structure is characterized using a laser Doppler vibrometer (LDV). A resonant frequency as high as 15 kHz is measured on some of the devices fabricated in this work. The higher resonant frequency translates into a faster switching speed in random access applications such as optical switching and higher resolution in single mirror free-space scanning applications.

**Figure 6:**
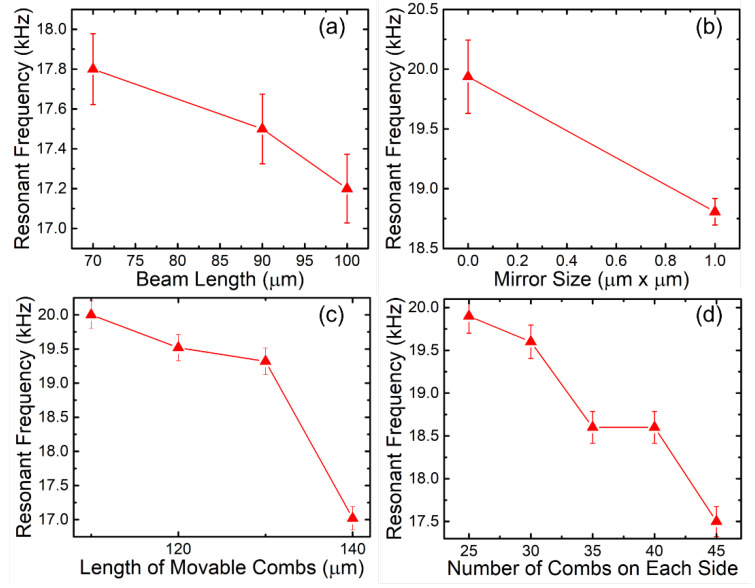
Resonant frequency as function of (**a**) beam length, (**b**) mirror size, (**c**) comb length and (**d**) the number of combs.

The characteristics of the 3D MEMS resonators in relation to the resonant frequency have been investigated in detail. First of all, the resonant frequency as a function of beam length is plotted in Figure 6a. From the measurement results, the resonant frequency decreases slightly with the increase in the beam length. Based on the Equations in (1)–(3), the relationship between resonant frequency and beam length can be derived as:

(4) $$f_{0}\propto \frac{1}{\sqrt{L}}$$

The Equation in (4) suggests that the resonant frequency is inversely proportional to the square root of beam length (*L*). This is also observed through the decreasing trend for the experimental results in Figure 6a. Therefore, both theoretical and experimental evaluation confirm that our device performs in agreement with the rotational mechanism of conventional 3D MEMS resonators. It can also be concluded that the beam length can be designed quantitatively according to the desired resonant frequency, as shorter beams give rise to higher frequency. 

The resonant frequency of the mirrors of various sizes is also measured by low-frequency signal sweeping. The mirror size is the dominant factor for the effective proof mass that affects the resonant frequency. The resonant frequency for two types of mirror sizes is shown in Figure 6b, and the results show clearly that the larger mirror size gives a lower resonant frequency that is achievable by the 3D comb drive. This is in agreement with Equation (3) given earlier, where the *W_m_* increases the torsional moment of inertia, $I_{\theta }$, subsequently reducing the resonant frequency $f_{0}$ in Equation (1). Further evaluation of the comb length, $l_{c}$, and number of comb fingers, N, in Figure 6c,d also reveals the same trend. These variables have a similar effect on $I_{\theta }$ and $f_{0}$, as given in Equations (1) and (3) too.

With the abovementioned observations, a finalized design is used to carry out the tilt angle measurements. As a larger range of motion for the tilt angle is preferred, the selection of the beam length must also be carefully chosen. A relationship between these variables is given here:

(5) $$\theta =\frac{T_{\theta }\cdot L}{K_{\theta }\cdot G}$$

**Figure 7:**
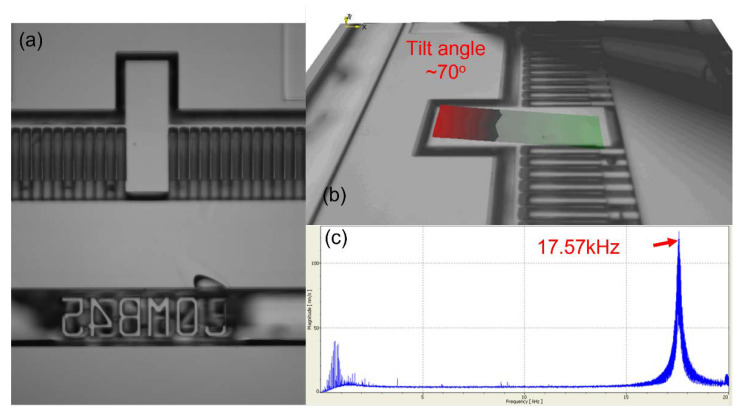
(**a**) SEM image of device (**b**) laser Doppler vibrometer results showing maximum tilt angle of 70° using AC bias of 2 V, (**c**) Fourier transform curve showing resonant frequency at 17.57 kHz.

This relationship suggests that the tilt angle (θ) is directly proportional to the beam length, representing an important tradeoff between resonant frequency and tilt angle in the design. The design parameters chosen are given in Table 1 with the results from the laser Doppler vibrometer given in Figure 7. A maximum tilt angle of 70° was experimentally obtained together with a resonant frequency of 17.57 kHz. In comparison with a conventional micromirror design using serpentine beams reported by Tsai et al. [23], the tilt angle measured here is much higher than theirs (50° tilt angle). Another CMOS–MEMS micromirror array fabricated through post-processing of CMOS chips developed by Cheng et al. [8] in 2017 also only gave a maximum tilt angle of 14.6° with a pull-in voltage of 49 V. With the results given here, it is conclusive that the device fabricated using the fabrication process here would thus be comparable with current state-of-the-art devices.

## 5. Conclusions and Summary

This paper presents a viable process development and characterization of a new CMOS-compatible process for the fabrication of high-aspect-ratio MEMS devices, which is based on the low-temperature plasma activated wafer bonding of two patterned wafers and DRIE bulk micromachining. A functioning test vehicle of a vertically stacked and out-of-plane comb structure is demonstrated. The vertical comb-drive structure is designed to be self-aligned in order to overcome any wafer-to-wafer alignment overlay. By systematic investigation of the resonant frequency, a series of data on the resonance the 3D MEMS devices with various design parameters is obtained and analyzed. A finalized design was also evaluated to produce a high tilt angle of 70° with an AC bias voltage of 2 V and a resonant frequency of 17.57 kHz comparable with current state-of-the-art devices. This process flow can be further refined for the future realization of 3D MEMS structures for a range of applications in sensing and actuation.

## Figures and Tables

**Table 1:** Design parameters for the comb-drive device.

Device Parameter	Nominal Value	Description
*L_m_*	300 µm	Mirror length
*W_m_*	100 µm	Mirror width
*l_c_*	120 µm	Comb finger length
*w_c_*	6 µm	Comb finger width
*g*	6 µm	Gap between comb fingers
*N*	60	The number of comb fingers
*L*	45 µm	Beam length
*W_b_*	4 µm	Beam width
*t*	40 µm	Thickness of beam, comb and mirror

## Data Availability

Not applicable.
